# Genome-Wide Analysis of the *AAAP* Gene Family in *Populus* and Functional Analysis of *PsAAAP21* in Root Growth and Amino Acid Transport

**DOI:** 10.3390/ijms24010624

**Published:** 2022-12-30

**Authors:** Jiujun Du, Changjian Du, Xiaolan Ge, Shuangshuang Wen, Xinglu Zhou, Lei Zhang, Jianjun Hu

**Affiliations:** 1 State Key Laboratory of Tree Genetics and Breeding, Key Laboratory of Tree Breeding and Cultivation of National Forestry and Grassland Administration, Research Institute of Forestry, Chinese Academy of Forestry, Beijing 100091, China; 2 Collaborative Innovation Center of Sustainable Forestry in Southern China, Nanjing Forestry University, Nanjing 210037, China

**Keywords:** *Populus*, *PtrAAAPs*, adventitious root, amino acid transport, QTL

## Abstract

The adventitious root (AR) is the basis for successful propagation by plant cuttings and tissue culture and is essential for maintaining the positive traits of a variety. Members of the amino acid/auxin permease (AAAP) gene family play indispensable roles in various plant metabolisms and have few studies on root growth and amino acid transport. In this study, with a systematic bioinformatics analysis of the *Populus* AAAP family, 83 *PtrAAAPs* were identified from *Populus trichocarpa* and grouped into 8 subfamilies. Subsequently, chromosomal distribution, genetic structure, cis-elements analysis, and expression pattern analysis of the AAAP family were performed and the potential gene *AAAP21* regulating root development was screened by combining the results of RNA-Seq and QTL mapping. *PsAAAP21* was proven as promoting root development by enhancing AR formation. Differentially expressed genes (DEGs) from RNA-seq results of overexpressing lines were enriched to multiple amino acid-related pathways, and the amino acid treatment to transgenic lines indicated that *PsAAAP21* regulated amino acid transport, including tyrosine, methionine, and arginine. Analysis of the AAAP gene family provided a theoretical basis for uncovering the functions of AAAP genes. The identification of *PsAAAP21* on root promotion and amino acid transport in *Populus* will help with breeding new woody plant species with strong rooting ability.

## 1. Introduction

The root is an essential plant organ and plays roles in absorption, support, transport, and synthesis; good root development is of great significance to the growth and development of plants. Moreover, the research on plant root development has become increasingly in-depth and has become a research hotspot [1,2,3,4]. Adventitious roots (ARs), which develop from non-root organs and dormant preformed meristem, or from adjacent vascular tissue cells in stems or leaves [5,6], form the structure of plant response to stress [7].

Nitrogen, an essential nutrient element for plant growth, is absorbed from the rhizosphere by the roots in the form of nitrates and ammonium salts and is stored in the form of amino acids and nitrogen-containing compounds [8]. Amino acids are important organic substances that maintain the normal growth and development of life and regulate the metabolism, structure, and biosynthesis of various compounds in eukaryotes [9,10]. Amino acids can be absorbed directly by roots as a nitrogen source [9], or evidently used as neurotransmitters and hormones for communication between cells and tissues [11,12]. The realization of these functions requires a specific transport system to transport amino acids between nucleus tissues, and amino acid/auxin permease (AAAP) is a protein that performs this function.

*AtAAP1*/*NAT2* was the first identified plant amino acid transporter and was found in *Arabidopsis thaliana* in 1993 [13]. The AAAP gene family is one of the largest amino acid transporter families and includes members from almost all eukaryotic organisms [14]. Aa_trans is the specific domain of the AAAP genes [15], and according to their structure, the AAAP family is further grouped into amino acid permease (AAP), lysine and histidine transporter (LHT), γ-aminobutyric acid transporter (GAT), auxin transporter (AUX), proline transporter (ProT), aromatic neutral amino acid transporter (ANT), and the amino acid transporter-like (comprising ATLa and ATLb) subfamilies [15,16]. To date, the functions of multiple *AAAPs* have been identified in several species, such as arabidopsis [15], tea tree [17], etc.

**Figure 1:**
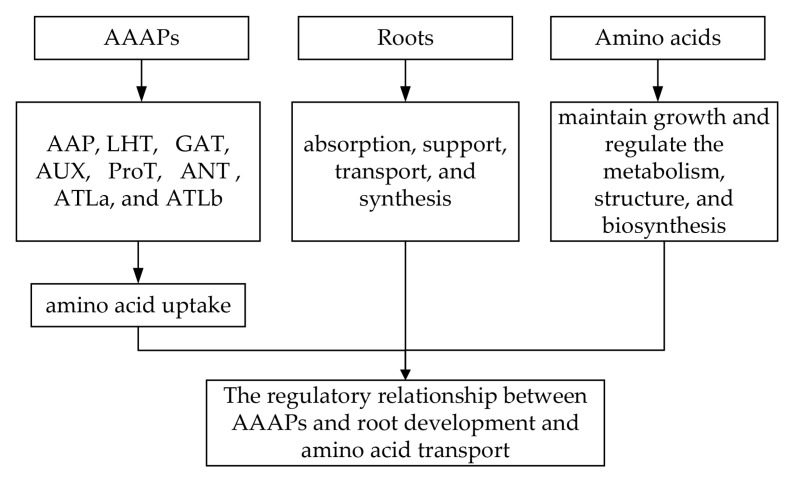
Research status and prospect of AAAPs, roots, and amino acids.

The research on the function of AAAP genes is gradually deepening. OsAAP6 is the positive regulator of rice grain protein content (GPC) promotes amino acid uptake by the root system and influences amino acid distribution [18]. Overexpression of *OsAAP4* increases rice tillering and grain yield as a result of enhancing the neutral amino acid concentrations of Val, Pro, Thr, and Leu. Exogenous Val or Pro significantly promotes the bud outgrowth and bud outgrowth overexpressing lines [19]. *OsAAP1* has similar functions in rice tillering and grain yield, the treatment with neutral amino acids can promote axillary bud outgrowth [20]. In arabidopsis, *AtAAP1* regulates root uptake and embryos loading neutral amino acids [9,21] (Figure 1).

Since the successful sequencing of *Populus trichocarpa*, *Populus* has become an important woody model plant [22,23,24]. Meanwhile, *Populus* is an important energy tree species. Improving the rooting status and nitrogen utilization capacity will help reduce the use of fossil fuels, protect the global environment, and provide a reference for the research of other woody plants [25].

Although *AAAP* genes have been identified and characterized in several plant species [15,16], there are no systematic reports of a comprehensive analysis and verification of root promotion and amino acid transport in *Populus*. Therefore, we aimed to identify and characterize the phylogenetic relationship and conserved domain architecture of *AAAP* genes in *Populus*, with the additional aim of selecting key genes to regulate poplar root traits and clarify the association between *PtrAAAPs*, root traits, and amino acid transport. This study provided a comprehensive bioinformatics analysis of AAAP genes in *Populus,* a basis for studying the regulation of root development and amino acid transport mediated by the AAAP gene *PsAAAP21*, and for cultivating new woody plant varieties with excellent root development and remarkable growth.

## 2. Results

### 2.1. Identification and Characteristics of PtrAAAPs

We identified 83 *PtrAAAPs* from *Populus trichocarpa* and *PtrAAAPs* encoding amino acid numbers ranging from 73 to 554. Among these, *PtrAAAP36* and *PtrAAAP63* encoded the least and most amino acid residues, respectively. Their relative molecular masses ranged from 7920.24 to 60390.81, with *PtrAAAP15* and *PtrAAAP63* being the smallest and largest, respectively. The theoretical pI ranged from 4.45 to 11.24, with *PtrAAAP5* and *PtrAAAP26* being the smallest and largest, respectively. The grand average of hydropathicity (GRAVY) ranged from 0 to 117.13, with *PtrAAAP15* and *PtrAAAP41* being the smallest and largest, respectively. Exon ranged from 1 to 11. The subcellular localization of *PtrAAAPs* was also predicted, with all *PtrAAAP* proteins localized to the cytoplasm, membrane, nucleus, vacuole, plasma membrane, and extracellular Only one signal peptide was predicted in *PtrAAAP35* for all *PtrAAAP* proteins (Table S1).

### 2.2. Chromosome Location and Evolutionary Analyses of PtrAAAPs

**Figure 2:**
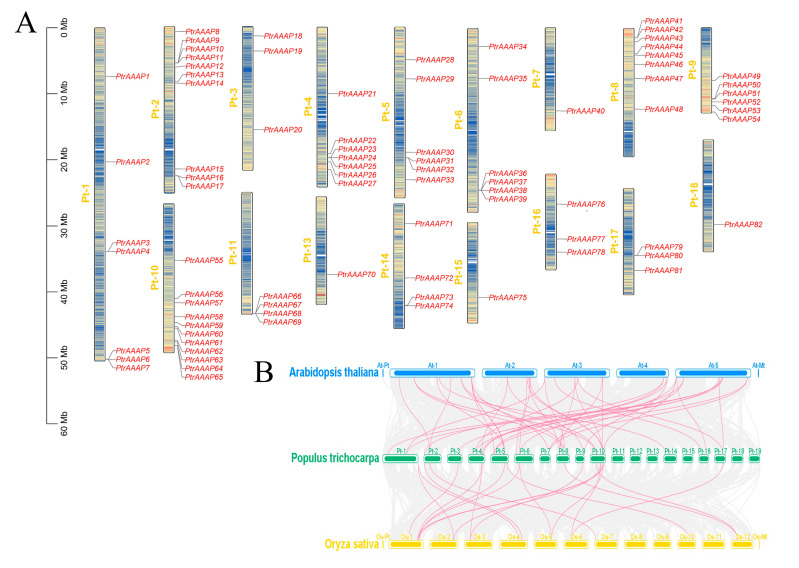
Chromosomal locations and collinearity analysis of the *PtrAAAPs*. (**A**) *PtrAAAPs* are marked on chromosomes; the scale bar on the left indicates the length of the chromosome Mb. (**B**) Collinearity relationship of AAAP genes among *Populus trichocarpa* (Pt), *Arabidopsis thaliana* (At), and *Oryza sativa* (Os). Identified collinear genes are linked by pink lines.

All *PtrAAAPs* were localized in chromosomes, except for *PtrAAAP83*, and *PtrAAAPs* were distributed on each chromosome with the exception of chromosomes 12 and 19. Chromosome 10 had the most *PtrAAAPs*, with 10, and chromosomes 7, 13, 15, and 18 had the fewest *PtrAAAPs*, all with only 1 (Figure 2A). Given that comparative co-linear mapping is useful for the study of evolutionary traits, comparative syntenic mapping was also established for *P. trichocarpa*, associated with *Arabidopsis thaliana*, and *Oryza sativa*, respectively (Figure 2B). Based on the results of the common lineage analysis, 44 and 20 orthologous pairs of genes were found in arabidopsis and rice, respectively (Table S2). *P. trichocarpa* was more closely related to arabidopsis. Two or more orthologous genes were found in arabidopsis for twelve *PtrAAAPs*, two or more orthologous genes were found in rice for four *PtrAAAPs*, and thirteen orthologous genes were found in both arabidopsis and rice for thirteen *PtrAAAPs*.

### 2.3. Phylogenetic Classification, Subfamily Division, and Structure of PtrAAAPs

**Figure 3:**
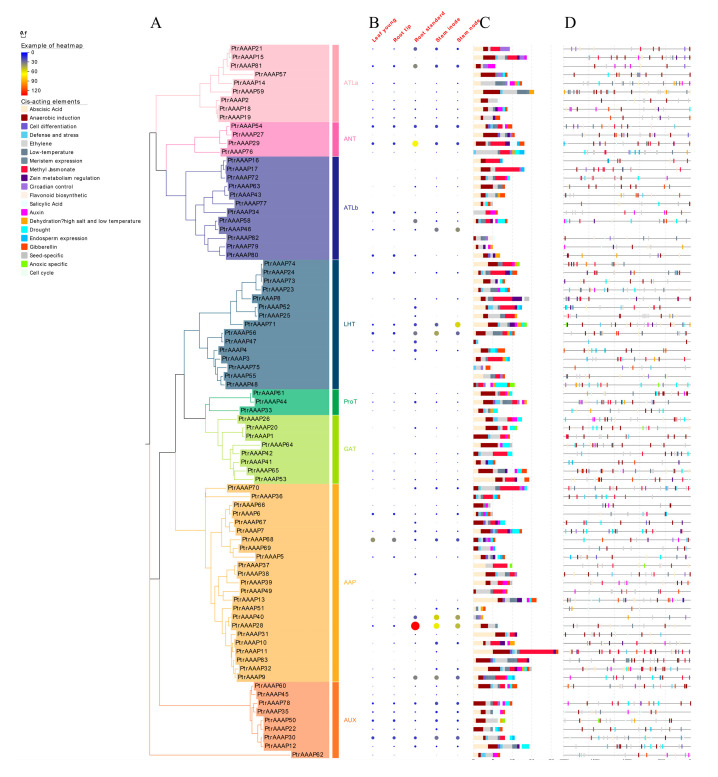
Phylogenetic tree of *PtrAAAPs*. (**A**) The name of each subfamily was marked on the outside of the phylogenetic tree. The *PtrAAAPs* were divided into ATLa, ANT, ATLb, LHT, ProT, GAT, AAP, and AUX 8 subfamilies. (**B**) The expression heatmap using phytozome online data. (**C**) Statistics of the cis-elements contained in the promoter region of each *PtrAAAP*. (**D**) Distribution of cis-elements of *PtrAAAPs* in chromosomes.

A phylogenetic tree of the *PtrAAAPs* was constructed by using the maximum likelihood estimate after aligning multiple protein sequences. *PtrAAAPs* were divided into eight subfamilies which were involved in LHT, ProT, GAT, AAP, ATLa, ANT, ATLb, and AUX. The number of genes in each subfamily also varies, with AAAP containing the largest number of *PtrAAAPs* (23) and ProT having the smallest number (3) (Figure 3A).

**Figure 4:**
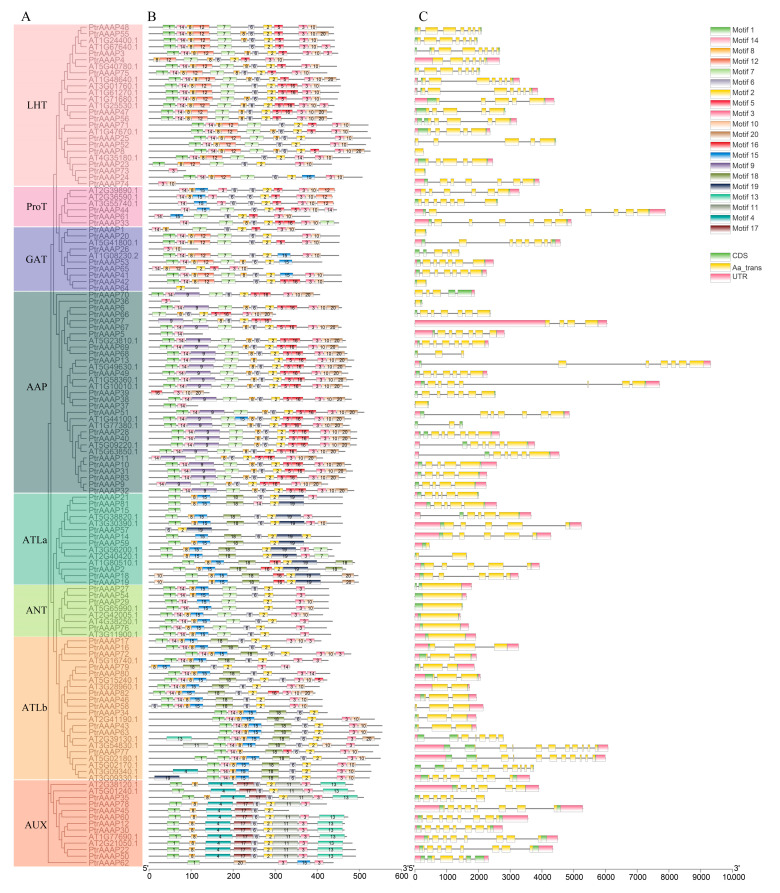
Phylogenetic and genetic structure analysis of *PtrAAAPs*. (**A**) Phylogenetic tree constructed using the maximum likelihood method and 83 *PtrAAAPs* protein sequences were divided into 8 subfamilies. (**B**) Distribution of motifs in *PtrAAAP* proteins, 20 motifs in total. (**C**) Structure of the *PtrAAAPs*, with the UTR in pink, the exon in green, the intron in the middle of the blank region, and the Aa_trans domain in yellow.

To further verify the accuracy of the phylogenetic tree, the AtAAAP protein sequences were constructed together with the PtrAAAP protein sequences, and each subfamily was distributed in *P*. *trichocarpa* and arabidopsis. At the same time, the motifs and structural features of each protein were marked (Figure 4), and each AAAP protein contained the Aa_trans domain. For each subfamily, the structures of the proteins were more conservative, the motifs were consistent, and the LHT and AAP subfamilies had the most complex structure and the largest number of proteins, which indicated that the phylogenetic tree and the results were accurate.

### 2.4. Identification of Cis-Elements of the PtrAAAPs Promoters

The cis-element in the 2000 bp region upstream of *PtrAAAPs* was identified, analyzed, and classified into three types: hormones, stress, and growth and metabolism. The hormone type contained the most cis-elements, followed by stress, and growth and metabolism contained the least. Among all the cis-elements, there were four types of them more than 170, abscisic acid (222), methyl jasmonate (172), ethylene (179), and anaerobic induction (220). Three of these, abscisic acid, methyl jasmonate, and ethylene, belonged to hormone type, one belonged to stress type, and none belonged to growth and metabolism type (Figure S1). The number of cis-elements in some *PtrAAAPs* was significantly different from other genes, for example, *PtrAAAP76* contained the most ethylene cis-element, and *PtrAAAP11* and *PtrAAAP17* contained the most methyl jasmonate cis-element. The number of cis-elements that differed significantly from other genes also indicated that they might play important roles in the response process of ethylene and methyl jasmonate (Figure 3C). Therefore, we concluded that *PtrAAAPs* play an important role in poplar hormone and stress response (Figure S1), of all the *PtrAAAPs*, the promoter region of *PtrAAAP59* contained the largest number of 25 cis-elements (Figure 3C,D).

### 2.5. Analysis of the Expression Pattern of PtrAAAPs

**Figure 5:**
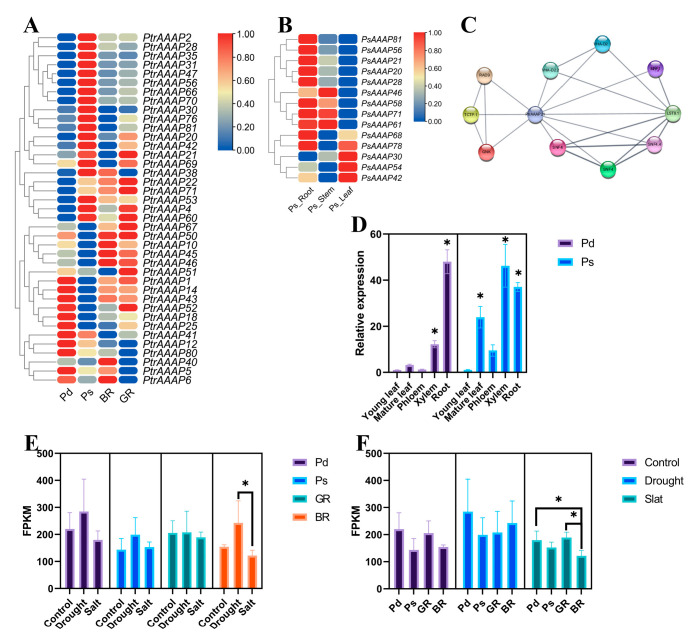
Expression pattern of *PtrAAAPs* and *AAAP21*. (**A**) *PtrAAAPs* selected from rooting RNA-seq, BR and GR represent average FPKM values of three bad rooting and three good rooting hybrid offspring, respectively. (**B**) Tissue-specific expression of selected *PsAAAPs* from *P. simonii* ‘Tongliao1’. RNA was extracted from roots, stems, and leaves, respectively. (**C**) Prediction of genes interacting with *PsAAAP21*. (**D**) Tissue-specific expression pattern of *P. deltoids* ‘Danhong’ and *P. simonii* ‘Tongliao1’. *AAAP21* expression from stress RNA-seq between parents and offspring and response to drought and salt, between (**E**) and between treatment. (**F**) GR: good rooting offspring F64, BR: bad rooting offspring F75. Pd: *Populus deltoides* ‘Danhong’, Ps: *Populus simonii* ‘Tongliao1’ and analysis based on ANOVA * *p* < 0.05.

The online website Phytozome was used to download tissue-specific expression data for *PtrAAAPs* (including root, stem, and leaf) and to plot the expression heatmap. To further validate the correctness of the tissue-specific expression data, 14 AAAP genes were selected, except for ANT and ProT subfamilies, and two genes from each subfamily were selected to determine their expression patterns in *Populus simonii* ‘Tongliao1’, and the expression pattern results were generally consistent with those of Phytozome, meanwhile, all 14 genes were highly expressed in the roots (Figure 5B).

The expression pattern of *PtrAAAPs* did not show a uniform pattern that genes with high expression were present in all tissues, of which *PtrAAAP68* was most highly expressed in young leaves and root tips, *PtrAAAP28* in root standard and stem node, and *PtrAAAP71* in stem node.

### 2.6. PtrAAAPs Function Analysis

Almost half of the *PtrAAAPs* (39/83) were found to be differentially expressed in the results of different individual root transcriptomes in the previous period. For these 39 genes, two expression patterns existed for higher and lower expression, with 15 and 24 genes, respectively, while the expression patterns of the 39 genes of the hybrid offspring and the parents essentially remained consistent [26]. These 39 AAAP genes were distributed in seven different subfamilies, with the largest number of AAP subfamilies, the least number of ANTs, and no genes from the ProT subfamily. In the same way, *PtrAAAPs* were searched in the results of QTL mapping for root and stem traits, and a total of three *PtrAAAPs* were found (Table S3), among of which *PtrAAAP21* and *PtrAAAP83* were correlated with root dry weight, and *PtrAAAP60* was correlated with leaf number trait. Furthermore, the qRT-PCR result of 14 *PtrAAAPs* showed the expression levels of *PsAAAP20*, *PsAAAP21*, *PsAAAP28*, and *PsAAAP78* were higher in roots (Figure 5B).

The regulation of root development by *AAAPs* has been reported in several papers, mostly in arabidopsis, but also in rice, pine, tea tree, ginseng, etc. In addition, several *AAAPs* have been reported to have regulatory effects on root development, including the translocation and uptake of amino acids, growth hormones, and nitrogen, improving plant response to stress and regulating root and root hair growth (Table 1). For *PtrAAAP21*, the expression level in the root was the highest (Figure 3B and Figure 5A,B), however, the expression pattern of *AAAP21* showed a regular pattern and *AAAP21* was highly expressed both in the xylem and roots of poplar, suggesting that *AAAP21* may regulate the growth and development of root and xylem, as well as the absorption and transport of nutrients (Figure 5D).

Analysis of the stress RNA-seq of parents and hybrid offspring showed that the expression of *AAAP21* was stable in the four poplar species and the expression of *P. deltoides* ‘Danhong’ (Pd) and good rooting offspring (GR) was higher than that of *P. simonii* ‘Tongliao1’ (Ps) and bad rooting offspring (BR). Moreover, *AAAP21* could respond to drought, but didn’t show significant reaction to salt, which indicated *AAAP21* was the positive factor for root development and drought response (Figure 5E,F). Several screening methods were used and *PsAAAP21* was selected, and it became of interest as to whether this gene regulates adventitious root development in *Populus*.

### 2.7. Regulatory Effect of PsAAAP21 on Root Development

**Figure 6:**
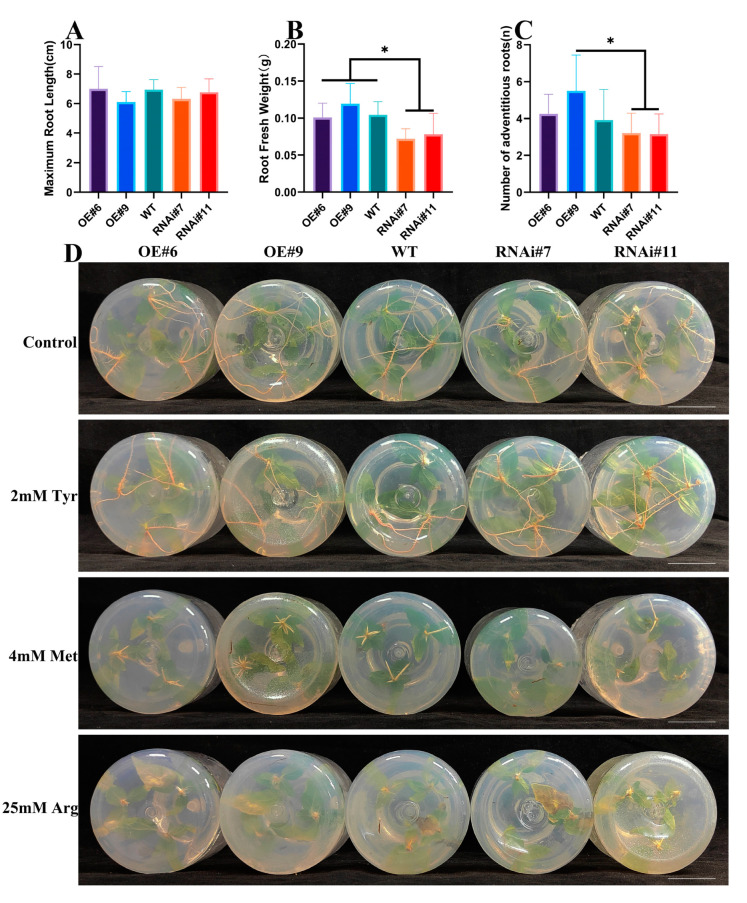
Phenotype determination. Phenotypic determination of maximum root length (**A**), root fresh weight (**B**), and the number of adventitious roots (**C**) for tissue cultured seedlings. Analysis based on ANOVA * *p* < 0.05. (**D**) The phenotype of transgenic lines treated with amino acids, scale = 3 cm.

Agrobacterium tumefaciens-mediated genetic transformation was utilized to obtain *PsAAAP21* overexpression and inhibition expression lines. Phenotypic analysis of *PsAAAP21* transgenic plants showed that *PsAAAP21* was a positive regulator of adventitious root (AR) development. Traits of root fresh weight and number of AR, but not maximum root of length, showed differences. The inhibited expression of *PsAAAP21* suppressed AR development, furthermore, this difference was caused by promoting the formation of AR (Figure 6A–C); on this basis, the growth of aboveground parts was also affected, which was consistent with the development of AR. This result showed that the promotion of AR will also improve the development of the aboveground parts. Therefore, the effect of *PsAAAP21* on plant biomass was also the direction of follow-up research (Figure S2).

### 2.8. Analysis of RNA-Seq with Hybrid Parents and Offspring

**Figure 7:**
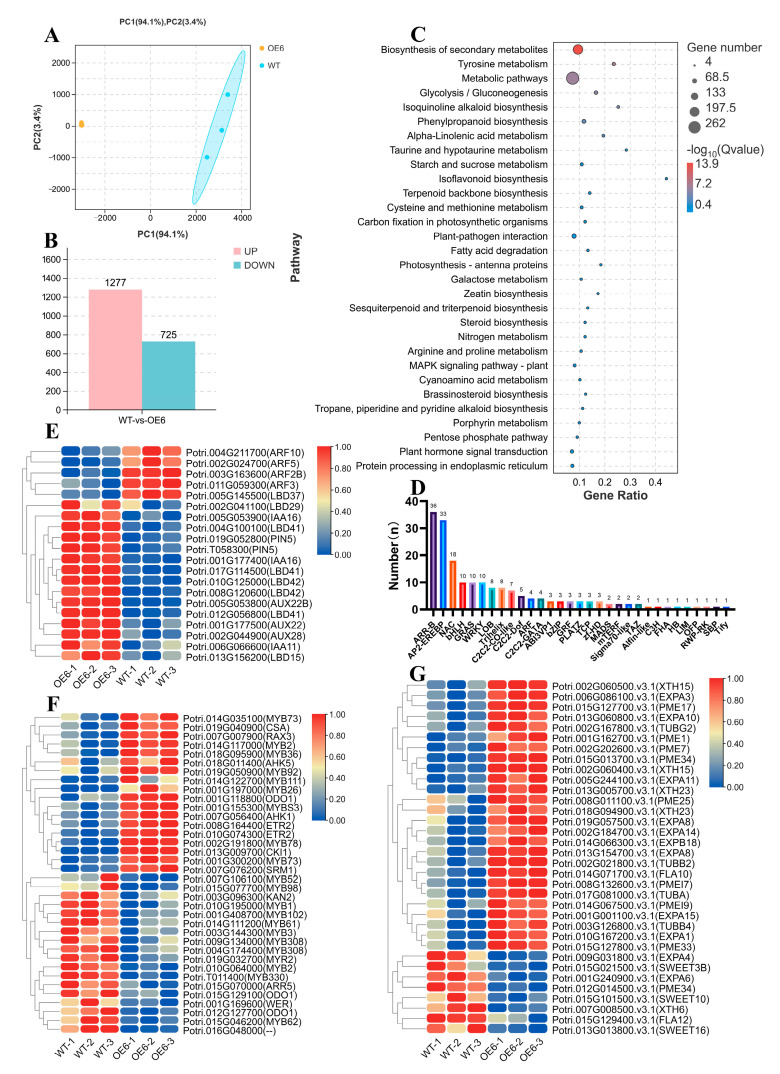
RNA-seq differentially expressed gene analysis. (**A**) Principal component analysis PCA of the expressed genes showing sample separation. Principal component 1 (PC1) and PC2 explaining 94.1 and 3.4% of the total variance, respectively. (**B**) The number of DEGs with upregulated genes in pink and downregulated genes in light blue. (**C**) Kyoto encyclopedia of genes and genomes KEGG. enrichment analysis of DEGs, node color represents -log10 transformed Qvalue—corrected *p*-Value, node size represents rich factor. (**D**) Number of transcription factors in DEGs. Heatmap of auxin (**E**) and cytokinin (**F**) related genes. (**G**) Heatmap of cell wall development and cell elongation related genes.

Compared with WT, *PsAAAP21* overexpression line RNA-seq results showed differences, 1277 genes were upregulated and 725 genes were downregulated, respectively (FDR ≤ 0.05, Fold change ≥ 2) (Figure 7B). Moreover, the Kyoto Encyclopedia of Genes and Genomes (KEGG) enrichment analysis of differentially expressed genes (DEGs) showed that pathway of biosynthesis of secondary metabolites and metabolic pathways contained the most number DEGs, moreover, the value of -log10 transformed Qvalue was highest with metabolic pathways, which denoted that *PsAAAP21* was involved in metabolic pathways (Figure 7C). The analysis of DEGs showed that it contained a number of transcription factors, including ARR-B, AP2-EREBP, ARF, TCP and so on, in which *ARRs* and *ARFs* are cytokinin and auxin pathway genes, respectively, and these two hormones are key hormones in AR initiation and development, meaning that *PsAAAP21* regulated AR development by affecting hormones. At the same time, we also noticed that genes *XTHs*, *EXPAs*, *PMEs*, *TUBs*, and *SWEETs,* associated with cell wall and cell elongation, also showed differences in expression, and these genes were also involved in the process of AR development.

### 2.9. Regulatory Effect of PsAAAP21 on Amino Acid Transport

**Figure 8:**
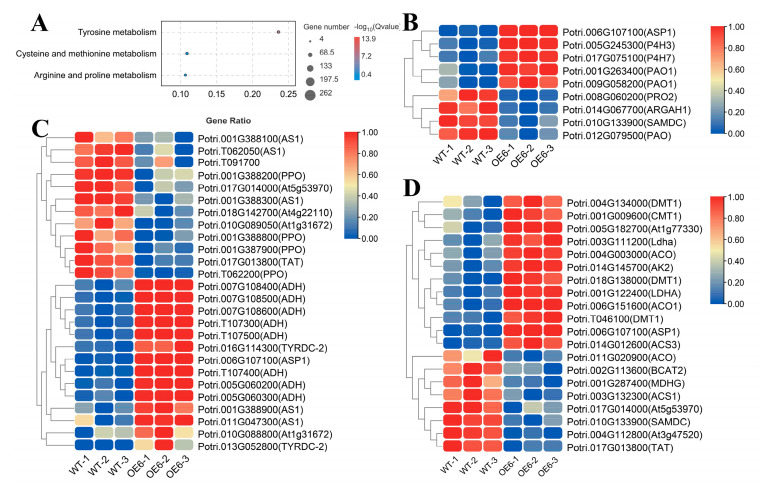
Results of RNA-Seq for amino acid transport. (**A**) Results for the Kyoto Encyclopedia of Genes and Genomes (KEGG) enrichment about amino acid. Heatmap for differentially expressed genes DEGs for the amino acid pathway of arginine and proline metabolism (**B**), tyrosine metabolism (**C**), and cysteine and methionine metabolism (**D**), respectively.

In the RNA-seq results of overexpressed strains (OE#6), the enrichment results of KEGG with DEGs showed that many DEGs were enriched to amino acid related pathways, including arginine and proline metabolism, tyrosine metabolism, and cystine and methionine metabolism, which contained 9, 26 and 20 genes, respectively. Meanwhile, there were two expression modes of positive and negative correlation (Figure 8A–D). Combined with the existing research results (amino acid determination results of phloem and xylem of hybrid parents and offspring), the correlation between *AAAP21* expression and amino acid content was analyzed. The expression of *AAAP21* was significantly correlated with tyrosine (Tyr) and arginine (Arg) content, especially with the opposite trend of Tyr content in phloem and xylem (−0.89 and 0.93) (Figure S3). In order to further verify the transport effect of *PsAAAP21* on amino acids, transgenic lines and WT plants were treated with amino acids, and the results showed that amino acids had inhibitory effects on plants. There was a greater inhibitory effect from 2 mM Tyr on overexpression plants and WT plants than on inhibited expression plants. However, there was a reduced inhibitory effect from 4 mM methionine (Met) on overexpression plants and WT plants than on inhibited expression plants, and this combined with the reduced inhibitory effect of 25 mM Arg on overexpression plants compared to WT and inhibited expression plants (Figure 6 and Figure S2).

## 3. Discussion

Amino acid/auxin permease (AAAP), a family of proteins that perform amino acid transport functions in plants, has been identified in several plants, including *Camellia sinensis*, *Medicago truncatula, Phyllostachys edulis*, and *Liriodendron chinense* [15,16,17,38]. Although the family has been reported, our knowledge of *Populus,* which important model and energy species and plays an important role in scientific research and production, is not comprehensive. [22,23,24,25,39]. As an important underground organ of plants, the good developmental status of roots contributes to the growth and development of plants [3]. Consequently, given the important role of *Populus* and the potential role of AAAP proteins on plant root development, the first systematic genome-wide analysis was performed in the *P. trichocarpa* genome and genetic transformation of *AAAP21* gene was performed to verify its regulation function on root development.

### 3.1. Genes Identified, Phylogenetic Classification, and Subfamily Division of PtrAAAPs

In this research, HMMER 3.0 was used to identify the *PtrAAAPs*, and 83 *PtrAAAP* genes were identified from *P. trichocarpa* (Table S1), the AAAP proteins of arabidopsis and rice were identified by the same method. *Populus* has 83 more AAAP genes than arabidopsis, *C*. *sinensis*, *M*. *truncatula, P*. *edulis*, which may be related to the at least three whole-genome duplication events in poplar and the subsequent multiple fragment duplication, tandem duplication, and transposition events (Figure 4) [15,16,17,23]. Subsequently, based on their structure, *PtrAAAPs* were divided into eight subfamilies, and the classification of each subfamily was consistent with arabidopsis (Figure 3) [15], which also indicated that AAAP family genes existed before the differentiation of *P. trichocarpa* and arabidopsis. By searching for orthologs of *P. trichocarpa* with arabidopsis and rice, 44 and 20 AAAP orthologs were present in the two species, respectively, indicating that arabidopsis is more closely related to *P. trichocarpa*. *P. trichocarpa* and arabidopsis as dicotyledons and rice as monocotyledons were also in good agreement with this result.

### 3.2. Structure and Evolution of PtrAAAPs

The conserved protein motifs and gene structures of *PtrAAAPs* were further investigated. As the important molecular basis for genes in the plant process of evolution, the structural features of genes play a crucial role in plant adaptation to environmental changes, which can be foundations to distinguish them from other gene families [40]. The subfamilies LHT and AAP of the *PtrAAAPs*’ eight subfamilies were more complex in terms of motif number, length, and structure, which also meant that they perform more complex functions in plants (Figure 4). As an important gene structure, introns participate in alternative splicing and control the speed of gene evolution [41]. The *PtrAAAPs* contained introns ranging from 0 to 10, and the number of introns contained in each subfamily of *PtrAAAPs* was similar (Figure 4), indicating that the genes in each subfamily play similar regulatory roles and corroborating the accuracy of the classification.

### 3.3. Regulatory Function on the Root of PsAAAP21 in Populus

**Figure 9:**
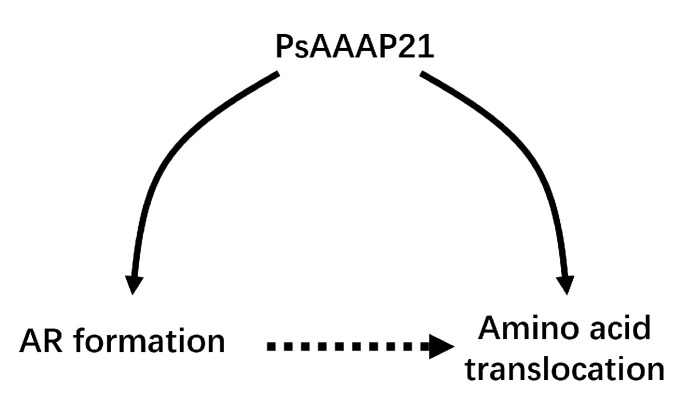
The mode of *PsAAAP21* regulates ARs and amino transport.

In the existing studies, in addition to *AAAPs* regulating amino acid absorption and transport, there are also reports on promoting root development [28,37]. AUX1 regulates root elongation by maintaining auxin accumulation, as a plant hormone, auxin regulates the development of AR [42]. AR development of *PsAAAP21* transgenic plants was changed, and *PsAAAP21* promoted root development by regulating the occurrence of AR (Figure 9), leading to the change of root dry weight. PPI forecast results showed that *PsAAAP21* interacted with *MAF1*, *LST8,* and *SNF4* (Figure 5C), which were confirmed to participate in root growth in existing research [43,44,45]. Although the results of yeast two-hybrid showed that PsAAAP21 did not interact with these three proteins, the reason for this result may be caused by differences between homologous genes, and in subsequent trials we will further clarify and look for the regulatory mode of *PsAAAP21* (Figure S4).

Based on results of RNA-seq, there were differences in the related gene expressions of auxin, cytokinin, and cell development. Auxin and cytokinin are important hormones for adventitious root initiation and development, auxin is involved in the initiation of adventitious roots, and cytokinin inhibits the initiation of adventitious roots but promotes the elongation of adventitious roots [46], and this antagonistic regulation has also been confirmed [47,48]. *PeARR12* inhibits AR formation by inhibiting the expression of *WOX5/11* and *PIN1/3* [49]. *PtRR13* is also a negative regulator of adventitious root development [50]. *LBDs* is an auxin pathway gene that regulates lateral root development and is accompanied by auxin concussion in this process [51,52]. PIN protein plays a key role in the auxin polar transport, and the response of roots to auxin gradient also needs to be mediated by AUX/IAA and ARF proteins [53,54]. *ARF7* and *ARF19* regulate the composition of root hair cell walls through *ERU* [55]. *PME* and *PMEI* are also necessary for the occurrence of lateral roots [51]. The expression levels of *PIN5* homologous genes and *LBDs* were increased in overexpressed lines, indicating that there were differences in the transport and synthesis of auxin in highly *PsAAAP21* expressed lines. Moreover, the expression levels of *XTHs*, *EXPAs* [56,57], *PMEs*, and *TUBs* were mostly increased in *PsAAAP21* overexpressed lines, therefore, *PsAAAP21* promoted the formation of adventitious roots by regulating auxin, and genes related to cell wall and cell elongation were also involved.

### 3.4. AAAPs Is Involved in Amino Acid Transport

Nitrogen is an important nutrient for plant growth and development. High nitrogen fertilizer can enable crops to obtain the highest yield. In the past few decades, to meet the population’s demand for food, the use of synthetic nitrogen fertilizer has increased significantly [58]. *AAAPs* are helpful for the transportation and utilization of amino acids, which is a source of nitrogen the soil, by plants [58,59]. *PsAAAP21* belonged to the AAAP gene family, which is involved in the transport of amino acids and auxin in plants [9,12,28,59,60]. Subcellular localization results showed that PsAAAP21 was localized to the endoplasmic reticulum (Figure S5), at the same time, transgenic lines and WT treated with amino acids showed different responses to amino acids, and the amino acid transport capacity of *PsAAAP21* was also proven (Figure 9). Since amino acids are a source of nitrogen in soil and there are precedents for *AAAPs* to improve nitrogen use efficiency [31], the amino acid transport capacity of *PsAAAP21* makes us think about whether *PsAAAP21* can affect the utilization of nitrogen by plants. Therefore, in subsequent experiments, transgenic lines will be treated with different concentrations of nitrogen sources to observe the response of plants to different concentrations of nitrogen, which can also provide a theoretical basis for the efficient utilization of nitrogen by plants.

## 4. Materials and Methods

### 4.1. Plant Materials

The *Populus simonii* ‘Tongliao1’ (Ps), used in this study was originally collected from a natural stand in Tongliao, Inner Mongolia Autonomous Region, and *Populus deltoides* ‘Danhong’ (Pd), a fast-growing and insect-resistant variety, were preserved in the nursery of Chinese Academy of Forestry. The 84K poplar (*Populus alba* × *Populus glandulosa*) was now preserved in the experimental site and tissue culture room of the Chinese Academy of Forestry under 2500 lx and 25 °C.

### 4.2. Identification, Characteristic, Chromosome Distribution, and Evolutionary Analysis of PtrAAAPs

After retrieving the Hidden Markov Model profiles of the Aa_trans domain (PF01490) from the Pfam database (http://pfam.xfam.org/, accessed on 19 February 2020) [61], the *Populus* bHLH proteins were identified with HMMER 3.0 (http://hmmer.janelia.org/, accessed on 19 February 2020) (E-Value < 0.01) from *P. trichocarpa* (Pt) (v3), downloaded from Ensembl Plant (http://plants.ensembl.org/index.html, accessed on 19 February 2020). Meanwhile, the *PtrAAAPs* were confirmed on SMART [62], pfam(http://pfam.xfam.org/, accessed on 19 February 2020) [61] and NCBI CDD (http://www.ncbi.nlm.nih.gov/cdd/, accessed on 19 February 2020). The *PtrAAAPs* characteristic and located information were identified with ProtParam (https://web.expasy.org/protparam/, accessed on 19 February 2020) [63] and TBtools [64] software, and the subcellular localization and signal peptides were analyzed with online database of Softberry (http://linux1.softberry.com/, accessed on 19 February 2020) and SignalP 4.1 (http://www.cbs.dtu.dk/services/SignalP-4.1/, accessed on 19 February 2020) [65]. TBtools [64] was used to finish synteny analysis with *Arabidopsis thaliana* and *Oryza sativa*.

### 4.3. Sequence Alignment and Phylogenetic Construction Tree of PtrAAAPs

Protein sequences of *PtrAAAPs*, extracted with Bio-Linux after being aligned by ClustalW with MEGA X (https://www.megasoftware.net/dload_win_gui, accessed on 19 February 2020), were divided into eight subfamilies according to their amino acid conservation. The Maximum likelihood estimate was used.

### 4.4. Structural Analysis of PtrAAAPs

MEME (http://meme-suite.org/, accessed on 19 February 2020) [66] was used to search for conserved motifs of *PtrAAAPs* protein sequences, the length and number were set to 6–50 and 20, and other parameters were the default values. The structure information of *PtrAAAPs* was extracted and TBtools [64] was used to draw *PtrAAAPs* structure map.

### 4.5. Analysis of Cis-Acting Elements and Protein-Protein Interaction Network of PtrAAAPs

PlantCARE [67] (http://bioinformatics.psb.ugent.be/webtools/plantcare/html/, accessed on 19 February 2020) of 2000 bp upstream sequences of the transcriptional start point for *PtrAAAPs* online website was used to identify cis-acting regulatory elements and then cis-acting regulatory elements were analyzed and classified after that. The *PsAAAP21* protein sequence was submitted to the online website String (https://string-db.org/, accessed on 19 February 2020) [68] to query and predict the potential regulatory effect of *PtrAAAPs* on root growth. The *P*. *trichocarpa* was selected as reference.

### 4.6. RNA Isolation and PtrAAAPs Expression Pattern Analysis

The expression pattern of *PtrAAAPs* was downloaded from Phytozome (https://phytozome-next.jgi.doe.gov/, (accessed on 19 February 2020). We analyzed the expression pattern of some genes from different subfamily in *P*. *simonii* ‘Tongliao1’ by qRT-PCR. RNAprep Pure Plant Kit (TIANGEN, Beijing, China) was used to extract the total RNA of roots, stems, and leaves of *P*. *simonii* ‘Tongliao1’ according to the manufacturer’s protocol, and then the total RNA was reverse transcribed into cDNA using a TIANScript II RT Kit (TIANGEN, Beijing, China). The *Actin* gene (*Potri.001G309500*) was used as the reference gene and the 2^−ΔΔCT^ method was used to analyze. All the experiments were performed with three replicates, primer information in Table S4.

### 4.7. Transcriptional Expression of PtrAAAPs during Root Development

Analysis and selection of the *PtrAAAP* genes in the differentially expressed genes in the RNA-seq of root development (transcriptome data comes from previous research by the research group of badly rooted and good rooted hybrid offspring), and qRT-PCR was used to analyze the expression of differential genes in the roots, stems, and leaves of *P*. *simonii* ‘Tongliao1’.

### 4.8. Gene Cloning, Vector Construction and Plant Transformation

In the previous research of the research group, *PtrAAAP21* was selected out based on the result of QTL mapping and RNA-seq of root growth of hybrid of *P. deltoides* ‘Danhong’ and *P. simonii* ‘Tongliao1’. The CDS sequence fragments of the *PtrAAAP21* gene was cloned from *P. simonii* ‘Tongliao1’ cDNA via specific primers, and the sequence was first cloned into the pDONR222 and then into pMDC32, utilizing BP and LR (Invitrogen, Shanghai, China). The method of Agrobacterium-mediated genetic transformation was taken to transform 84K poplar. *PsAAAP21* and *LST8.1*, *SNF4*, and *MAF4*, cloned from 84K poplar, were cloned into pGBDT7 and pGADT7-rec1, respectively.

### 4.9. Determination of Physiological Indexes of PsAAAP21 Overexpression Plants

Transgenic 84K poplar lines (OE#6, OE#9, RNAi#7, and RNAi#11) and wild-type 84K poplar (WT) were cultured in the tissue culture room of the Chinese Academy of Forestry, and the growth phenotype was determined after one month of growth.

### 4.10. RNA-Seq for Stress Treatment Hybrid Parents and Offspring and PsAAAP21 Overexpression Line

Hybrid parents and offspring cutting seedlings with consistent growth were selected for treatment. The water content in the drought treatment group was controlled to be 60–70% of the maximum water holding capacity, the salt stress group was treated with 150 mM NaCl, and the control group was treated with water, and the water content of salt stress and control was 100%. After one month of growth, root tips were collected, frozen in liquid nitrogen and sent to Biomarker Technologies Co, Ltd. (Beijing, China) for RNA-seq, three replicates per line. The 150 bp paired-end reads were generated on the Illumina NovaSeq 6000 platform and *P. trichocarpa* v4.0 was used as reference (FDR ≤ 0.05, Fold change ≥ 2).

Roots from one-month seedlings of *PsAAAP21* overexpression line #6 and wild type tissue culture were sampled, frozen in liquid nitrogen and sent to GENEDENOVO Biotechnology Co., Ltd. (Guangzhou, China) for RNA-seq, three replicates per line, and each replicate was pooled from three plants. The 150 bp paired-end reads were generated on the Illumina NovaSeq 6000 platform, and *P. trichocarpa* v3.1 was used as reference (FDR ≤ 0.05, Fold change ≥ 2).

### 4.11. Amino Acid Treatment

In order to verify the amino acid transport function of *PsAAAP21*, wild-type 84K poplar (WT) and transgenic plants (OE#6, OE#9, RNAi#7, and RNAi#11) and a variety of amino acids were selected for verification, including tyrosine (Tyr), methionine (Met), and arginine (Arg), their concentrations were 2 mM, 4 mM, and 25 mM, respectively, and the amino acid solution was added to sterilized 1/2 MS medium after filtration sterilization. The culture conditions were 25 °C and 2500 lx with three replicates at least per line.

## 5. Conclusions

In this study, HMMER search was used to identy *PtrAAAPs* in *Populus* and *PsAAAP21* was identified to integrate adventitious roots development and amino acid transport. Consequently, 83 *PtrAAAPs* were identified and characterized in *Populus*, and systematic bioinformatics analysis was performed and the possible regulatory role of *PtrAAAPs* was predicted. Combining RNA-seq and QTL mapping results, *PsAAAP21* was screened and cloned from *P. simonii* ‘Tongliao1’. The phenotype of transgenic plants showed that *PsAAAP21* promoted adventitious root development by regulating auxin. The results of the RNA-seq and amino acid content determination showed that *PsAAAP21* was related to amino acid transport, which was proved by amino acid treatment experiment. This study helps us to understand the AAAP gene family of *Populus* and uncover the theoretical basis for improving rooting and growth traits. Finally, we could preserve excellent traits of woody plants by using AAAP gene family and increase plant nitrogen utilization by the *AAAPs* function on amino acid transport.

## Figures and Tables

**Table 1:** *PtrAAAPs* regulate root development.

Gene ID	Species	Description
*OsAAP6*	*Oryza sativa*	Promoted amino acid uptake by the root [18].
*OsAAAPs*	*Oryza sativa*	FA-induced gene expression of AAAP transporters may contribute to detoxicification of the autotoxin [27].
*AUX1*	*Arabidopsis thaliana*	Restoration of root response to auxin [12].
*AUX1*	*Arabidopsis thaliana*	Maintained root elongation through maintenance of the auxin accumulation in root tips [28].
*AtAAP1*	*Arabidopsis thaliana*	Regulated roots uptake neutral amino acids [9].
*AtAAP3, AtAAP6*	*Arabidopsis thaliana*	The transport of amino acids by *AAP3* and *AAP6* was important for nematode infection [29], *AAP3* was related to root nitrogen uptake function [30].
*AtLHT1*	*Arabidopsis thaliana*	The capacity for amino acid uptake, and thus nitrogen use efficiency, was increased severalfold by *LHT1* overexpression [31].
*AtProT2*	*Arabidopsis thaliana*	Influencing nitrogen distribution during water stress [32].
*AAAP12*	*Vicia narbonensis*	Improved plant uptake and allocation of carbon and nitrogen [33].
*CsAAAPs*	*Camellia sinensis*	Related to theanine transport [34].
*CsAAP1*	*Camellia sinensis*	*CsAAP1* expression in the root was highly correlated with root-to-bud transport of theanine [35].
*PpAAP1*	*Pinus pinaster*	High-affinity arginine transporter in maritime pine [36].
*PgLHT*	*Panax ginseng*	Promoted the development of plants, especially root hair [37].

## Data Availability

The raw sequence data reported in this paper have been deposited in the Genome Sequence Archive in National Genomics Data Center, China, and the National Center for Bioinformation/Beijing Institute of Genomics, Chinese Academy of Sciences (GSA: CRA007914 and CRA008053) that are publicly accessible at https://ngdc.cncb.ac.cn/gsa (accessed on 2 September 2022).

## Supplementary Materials

The following supporting information can be downloaded at: https://www.mdpi.com/article/10.3390/ijms24010624/s1.
