# Supplementary material for: Genome-Wide Analysis of the AAAP Gene Family in Populus and Functional Analysis of PsAAAP21 in Root Growth and Amino Acid Transport
Source: Int J Mol Sci. 2022 Dec 30;24(1):624. doi: 10.3390/ijms24010624 (PMC9820651; doi:10.3390/ijms24010624)
Supplement: Supplementary file 1 [file ijms-24-00624-s001.zip › Supplementary materials.pdf]

Supplementary Table

Table S1 Information of *PtrAAAP* genes.

Table S2. Collinearity relationship of AAAP genes among *A. thaliana*, *P. trichocarpa*, and *O. sativa*.

Table S3. Quantitative trait locus (QTL) information for the investigated traits in the F1 population of *PtrAAAPs*.

Table S4. Primer sequences of *PtrAAAPs* for qPCR analysis and vector construction.

Supplementary Figure

Figure S1. Statistics of cis-acting elements of *PsAAAPs*, the cis-acting elements in the 2000 bp region upstream of *PtrAAAPs* are divided into 3 groups according to their function, the number of each cis-acting element of *PtrAAAPs* was marked in figure.

Figure S2. Stem phenotype of transgenic lines treated with amino acids.

Figure S3. Correlation analysis of *AAAP21* expression and amino acid content of hybrid parents. X_ represents xylem and *p*_ represents phloem. (* $P< 0.05$, ** $P < 0.01$, *** $P < 0.001$).

Figure S4. Yeast two-hybrid assay.

Figure S5. Subcellular localization of *PsAAAP21*. OsART1 and HDEL are markers of the nucleus and endoplasmic reticulum, respectively.
