# Supplementary figures and images for: Genome-Wide Analysis of the AAAP Gene Family in Populus and Functional Analysis of PsAAAP21 in Root Growth and Amino Acid Transport
Source: Int J Mol Sci. 2022 Dec 30;24(1):624. doi: 10.3390/ijms24010624 (PMC9820651; doi:10.3390/ijms24010624)

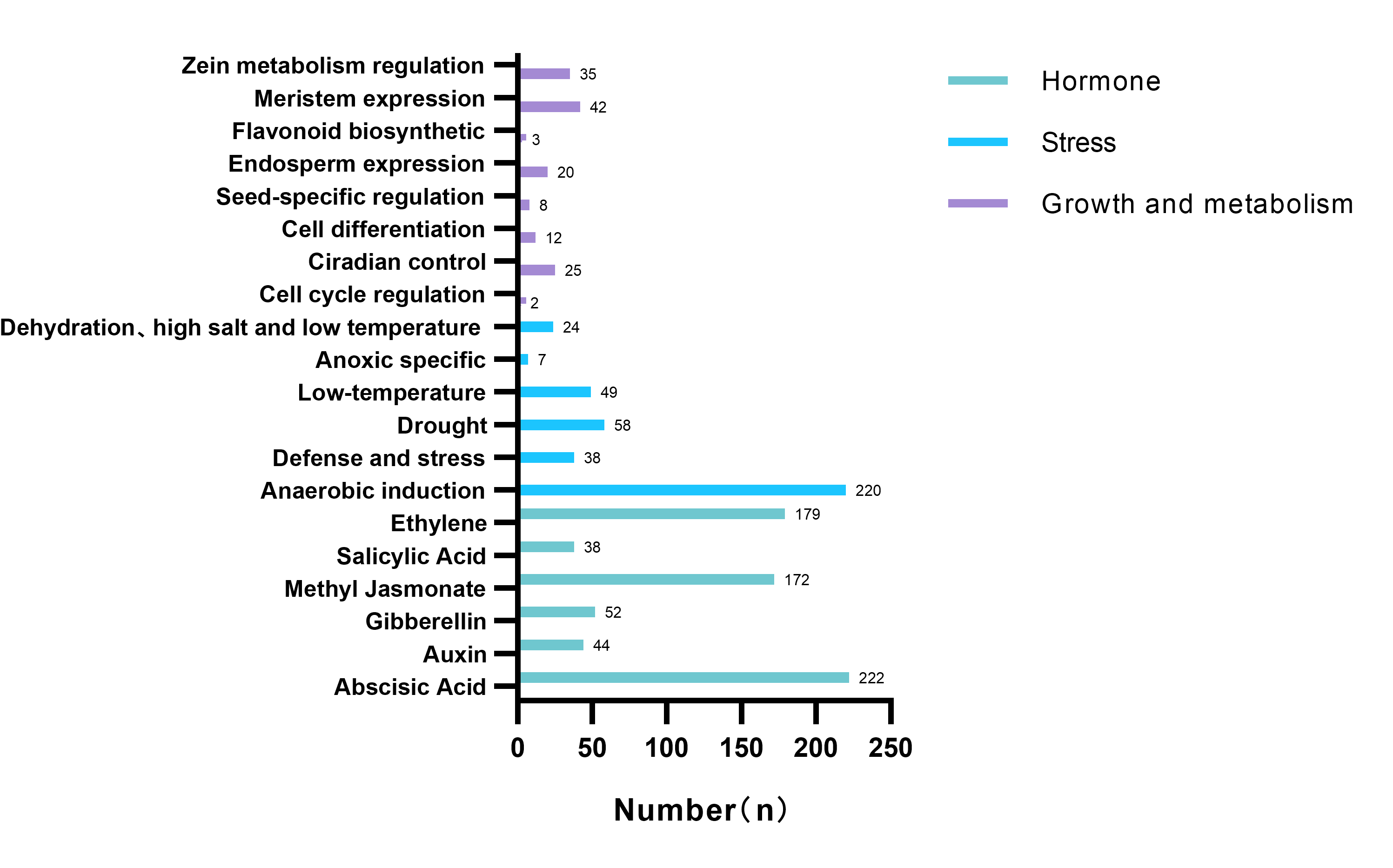

Supplement: Supplementary file 1 [file ijms-24-00624-s001.zip › Figure S1.tif]

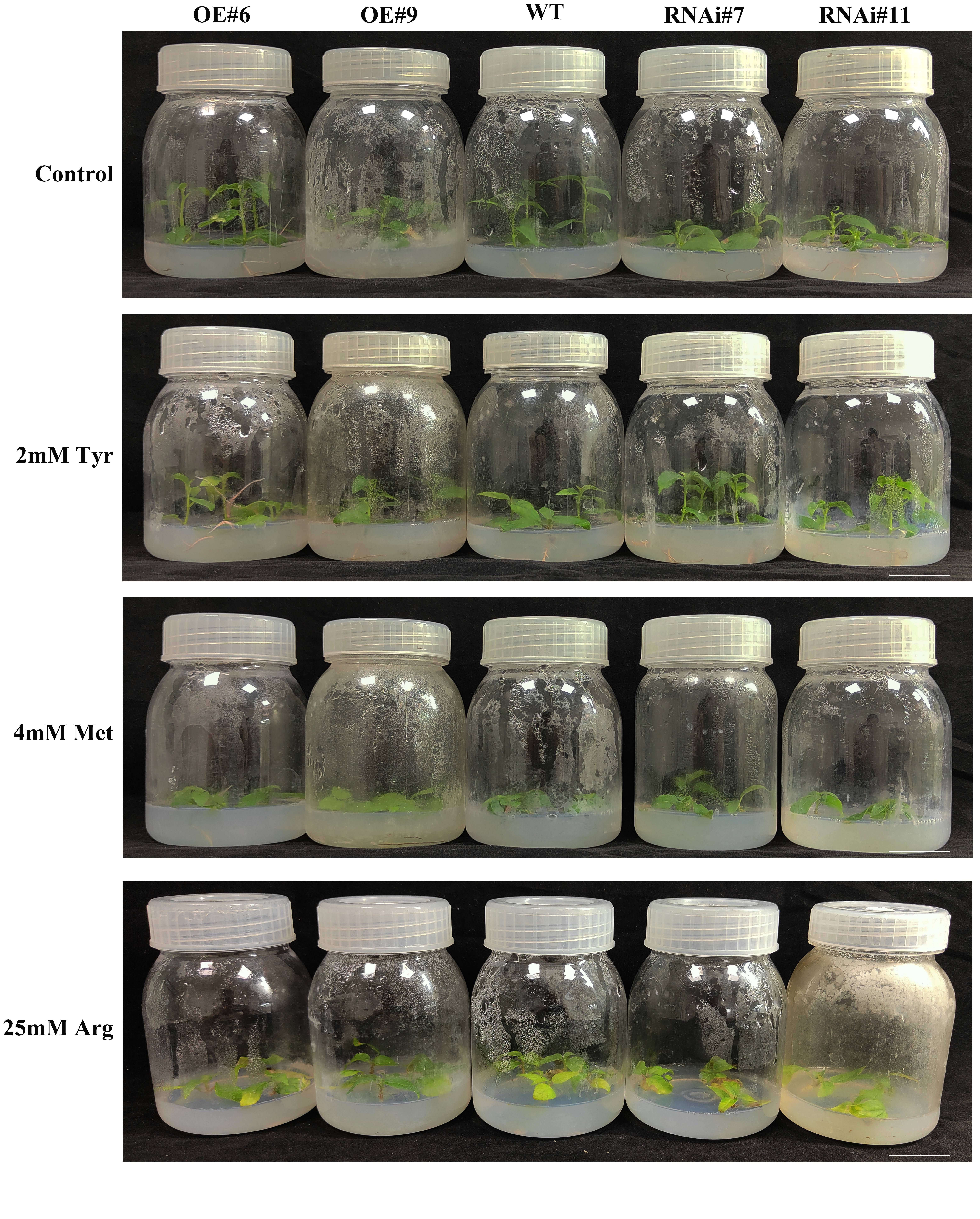

Supplement: Supplementary file 1 [file ijms-24-00624-s001.zip › Figure S2.jpg]

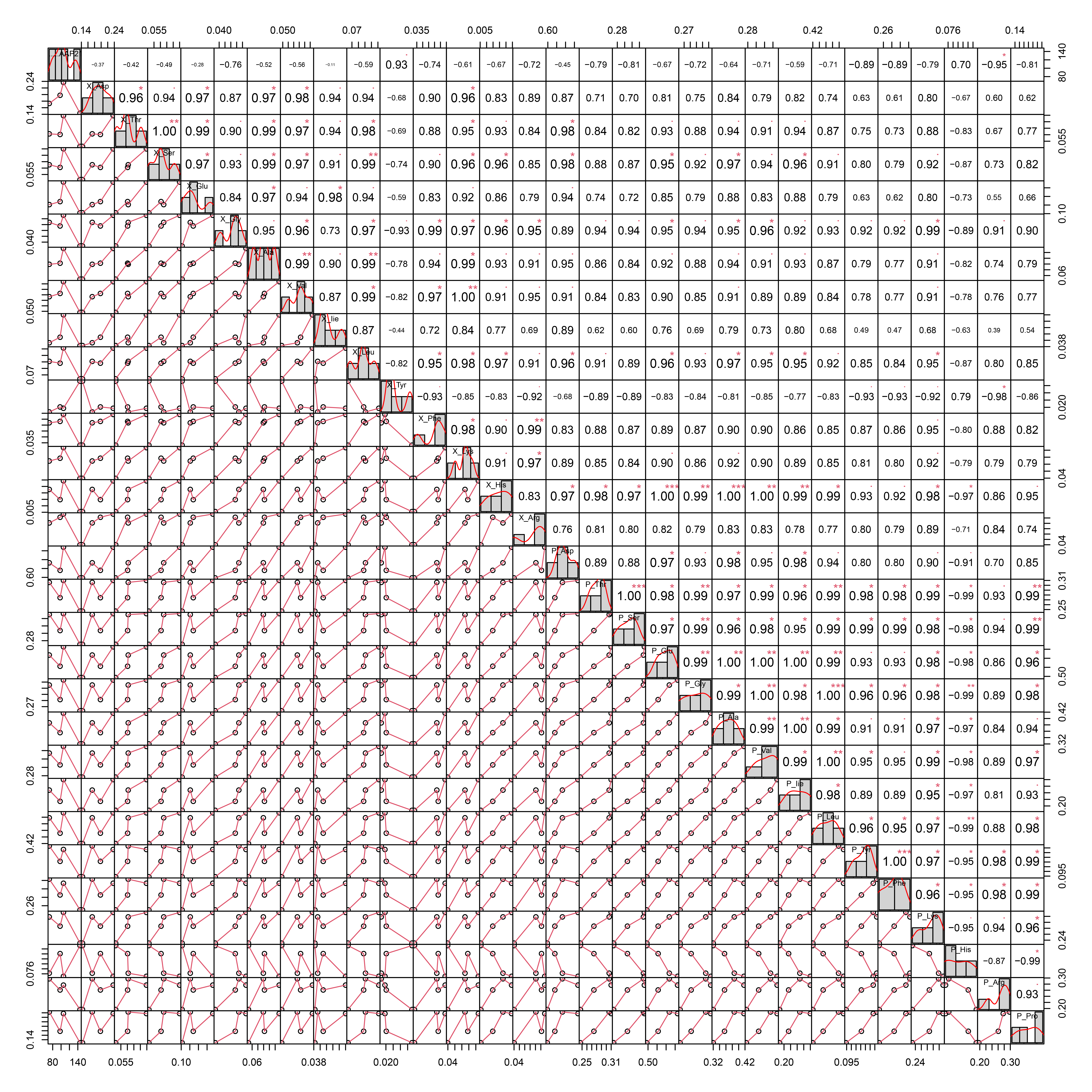

Supplement: Supplementary file 1 [file ijms-24-00624-s001.zip › Figure S3. Correlation analysis of AAAP21 expression and amino acid content of hybrid parents.jpg]

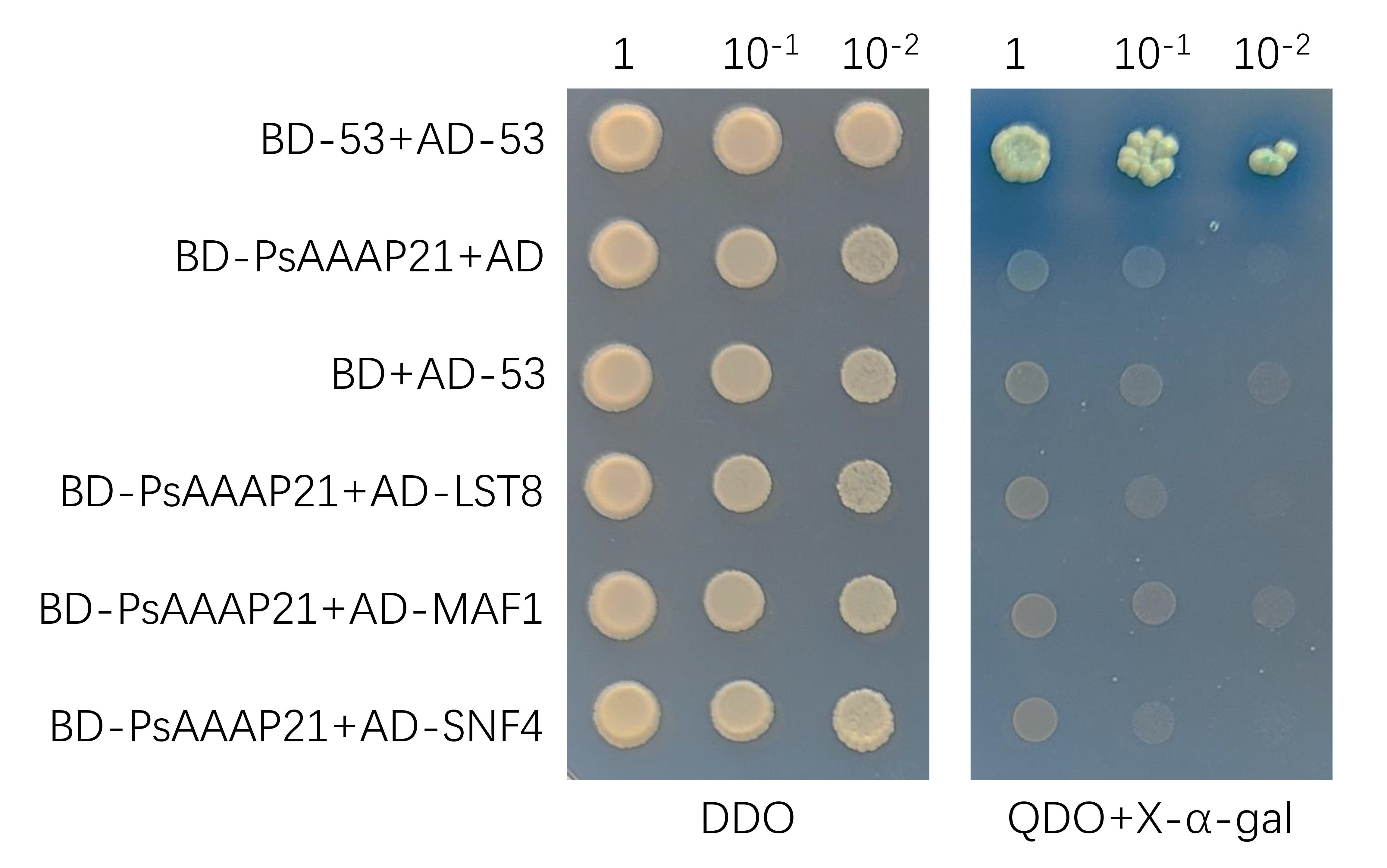

Supplement: Supplementary file 1 [file ijms-24-00624-s001.zip › Figure S4.jpg]

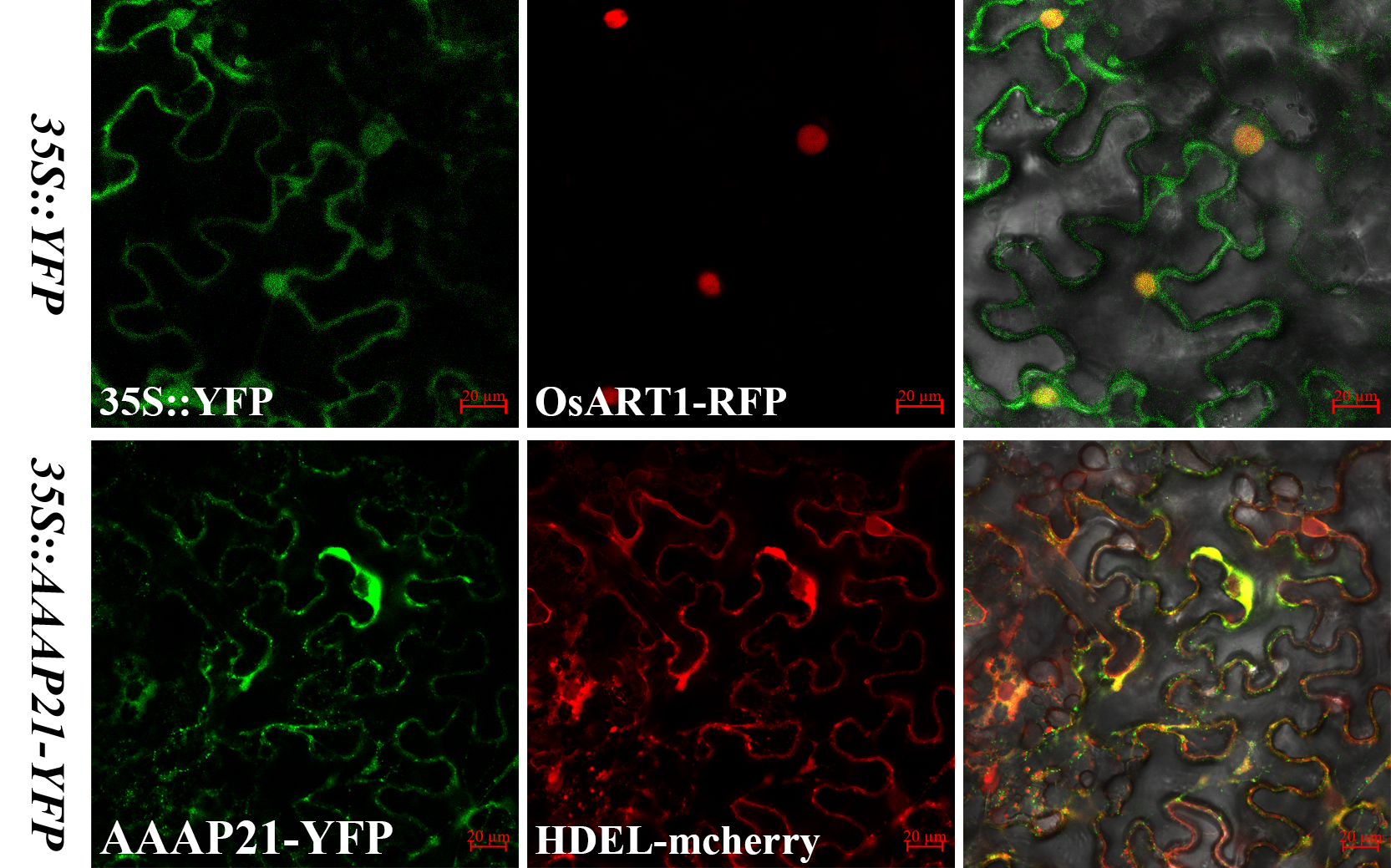

Supplement: Supplementary file 1 [file ijms-24-00624-s001.zip › Figure S5.jpg]
